# Supplementary material for: A principle-based framework to determine countries’ fair warming contributions to the Paris Agreement
Source: Nat Commun. 2025 Jan 26;16:1043. doi: 10.1038/s41467-025-56397-6 (PMC11770098; doi:10.1038/s41467-025-56397-6)
Supplement: Supplementary file 1 — Supplementary Information [file 41467_2025_56397_MOESM1_ESM.pdf]

A principle-based framework to determine countries' fair warming contributions to the Paris Agreement

Mingyu Li [1,2], Setu Pelz [3], Robin Lamboll [2], Can Wang [1], Joeri Rogelj [2,3,4 *]

1 School of Environment, *Tsinghua University, Beijing, China*

2 Centre for Environmental Policy, *Imperial College London, London, UK*

3 Energy, Climate and Environment Program, *International Institute for Applied Systems Analysis, Laxenburg, Austria*

4 Grantham Institute – Climate Change and Environment, *Imperial College London, London, UK*

* Corresponding author: j.rogelj@imperial.ac.uk

This file includes:

Supplementary Notes 1 to 9

Supplementary Fig. 1 to 17

Supplementary Table 1 to 4

Supplementary References

Contents

Supplementary Notes 4

Supplementary Note 1: Conceptual schematic of the resolution of countries' unequal historical warming contributions by 2050 through fair warming allocations 4

Supplementary Note 2: Influence of currency conversion methods on GDP 4

Supplementary Note 3: Influence of considering colonial powers in calculating historical fossil fuel sales 4

Supplementary Note 4: Influence of different starting year of historical warming contribution 4

Supplementary Note 5: Sensitivity to degree to which a country's allocation is changed as a function of differences in driving indicators 5

Supplementary Note 6: Influence of different desired peak global warming target 5

Supplementary Note 7: Sensitivity to population projection 5

Supplementary Note 8: Illustration of alternative country categorization method 6

Supplementary Note 9: Description of data sources and data preparation process 7

Supplementary Figures 8

Supplementary Fig. 1 Conceptual schematic of the resolution of countries' unequal historical warming contributions by 2050 through fair warming allocations. 8

Supplementary Fig. 2 Influence of GDP definition on CO_2 warming equivalent budgets. 8

Supplementary Fig. 3 Influence of apportioning fossil fuel sales during colonial occupation to colonizers 9

Supplementary Fig. 4 Remaining (2021-2050) CO_2 warming equivalent budgets per capita, compared between different starting years of historical warming contribution 10

Supplementary Fig. 5 Total (1850-2050), historical (1850-2021), and remaining (2022-2050) CO_2 warming equivalent budgets per capita by country (starting year of historical warming contribution of 1990) 11

Supplementary Fig. 6 The uncertainty in remaining (2022-2050) CO_2 warming equivalent budgets for major countries, with various maximum adjustment extent of driving indicators 12

Supplementary Fig. 7 Total (1850-2050), historical (1850-2021), and remaining (2022-2050) CO_2 warming equivalent budgets per capita by country (adjustment extent of 75%) 13

Supplementary Fig. 8 The uncertainty in remaining (2022-2050) CO_2 warming equivalent budgets for major countries, with various maximum adjustment extent of driving indicators 14

Supplementary Fig. 9 Remaining (2022-2050) CO_2 warming equivalent budgets per capita, compared between different desired peak global warming target 15

Supplementary Fig. 10 Total (1850-2050), historical (1850-2021), and remaining (2021-2050) CO_2 warming equivalent budgets per capita by country (desired peak global warming target of 1.7℃). ... 16

Supplementary Fig. 11 Total (1850-2050), historical (1850-2021), and remaining (2021-2050) CO_2 warming equivalent budgets per capita by country (desired peak global warming target of 2℃) ... 17

Supplementary Fig. 12 Influence of population projection on CO_2 warming equivalent budgets ... 18

Supplementary Fig. 13 The uncertainty in remaining (2022-2050) CO_2 warming equivalent budgets for major countries, with various population projections ... 18

Supplementary Fig. 14 Total (1850-2050), historical (1850-2021), and remaining (2022-2050) CO_2 warming equivalent budgets per capita by country. ... 19

Supplementary Fig. 15 Total (1850-2050), historical (1850-2021), and remaining (2022-2050) CO_2 warming equivalent budgets per capita by country ... 20

Supplementary Fig. 16 Annual fair CO_2 warming equivalent emission budgets against deepest available domestic reduction (DADR) pathways ... 22

Supplementary Fig. 17 The depletion of remaining CO_2 warming equivalent emission budgets over time. ... 22

Supplementary Tables ... 24

Supplementary Table 1 Data sources used in this study ... 24

Supplementary Table 2 Data sources for 10-year bond yield values per country ... 24

Supplementary Table 3 Normative, methodological and physical factors considered and their parameter settings ... 25

Supplementary Table 4 Warming masked by aerosol at various percentiles and consistent with a set of peak levels of global warming ... 26

Supplementary Notes

Supplementary Note 1: Conceptual schematic of the resolution of countries' unequal historical warming contributions by 2050 through fair warming allocations

Historical warming-equivalent CO_2 budget per capita is unevenly distributed among countries, especially between country groups. We force this unequal historical warming contributions to resolve by 2050. After 2050, all countries' fair shares turn to 0 $GtCO_2$-we per capita per year (Supplementary Fig. 1).

Supplementary Note 2: Influence of currency conversion methods on GDP

While GDP is one representation of a country's ability to pay, methods to estimate GDP exchange rates remain subject to debate[1]. Estimating GDP using purchasing power parity (PPP) tends to overestimate the capability of developing countries, especially when related to international-sourced products or finance. On the other hand, estimating using market exchange rates (MER) may not properly reflect the balance of a country between mitigation and other goods.

In the central case, we adopt GDP in PPP. Here we compare the results of both currency conversion methods on GDP (Supplementary Fig. 2). Our calculation indicates the influence of different GDP conversion methods is generally negligible on the remaining budget.

Supplementary Note 3: Influence of considering colonial powers in calculating historical fossil fuel sales

In the central case, we take into consideration countries' colonial histories when estimating fossil fuel sales. During colonisation, a colonial country seized wealth of colonized countries. Here we assign responsibility for the emissions during this period to the colonizing country as it is assumed that all benefits are controlled and enjoyed by the colonizing country. This is a first-order proxy as some assets like railways and roads could have been built and handed over later, which would transfer some of the benefits to the colonized country, although we consider this to be a secondary effect only.

We compare the results when historical fossil fuel sales are redistributed to the colonizers and when they are not. The overall effects are limited. 19 countries have a relative change of fossil fuel sales exceeding 1%, mainly African countries and major European colonial empires, including Madagascar (-82%), Morocco (-41%), Zimbabwe (-35%), Brunei Darussalam (-33%), Portugal (+610%), the UK (+10%), Netherlands (+4%), France (+3%). Yet, the influence on the quantifications of the remaining budgets is nevertheless only minor (Supplementary Fig. 3).

Supplementary Note 4: Influence of different starting year of historical warming contribution

In the central case, we adopt 1850 as the starting year of historical warming contribution. We explore an alternative of 1990 (Supplementary Fig. 4).

Here we present alternative versions of Figure 2 of the main text but with starting year of historical warming contribution of 1990 (Supplementary Fig. 5).

Supplementary Note 5: Sensitivity to degree to which a country's allocation is changed as a function of differences in driving indicators

In the central case, we adopt 50% as the maximum degree to which a country's allocation is changed as a function of differences in driving indicators (adjustment extent). We explore an alternative of 25% and 75%. Assuming a 100% adjustment extent, countries with extreme values will get double or zero allowable total warming budget compared to Interpretation I.

The distinction between country groups is normally enlarged with larger adjustment extent (Supplementary Fig. 6). With larger adjustment, country ranking could be reversed. Still, the effect seems to be moderate on reversing the country ranking.

Here we present alternative versions of Figure 2 of the main text but with an indicator adjustment extent of 75% showing strong visual alignment and robustness of the qualitative insights of our study (Supplementary Fig. 7).

Supplementary Note 6: Influence of different desired peak global warming target

In the central case, we adopt 1.5°C as the desired peak global warming target. We explore an alternative of 1.7°C and 2°C (Supplementary Fig. 8).

We further have a deeper look in to the comparation of the two extreme desired peak global warming targets of 1.5°C and 2°C (Supplementary Fig. 9).

Here we present an alternative to Figure 2 in the main text with a desired peak global warming target of 1.7°C (Supplementary Fig. 10).

Here we present an alternative to Figure 2 in the main text with a desired peak global warming target of 2°C (Supplementary Fig. 11).

Supplementary Note 7: Sensitivity to population projection

For population data, we followed United Nations medium scenario projection in the main text, a scenario with medium fertility, medium mortality, and medium international migration. We also consider low fertility and high fertility population projections as sensitivity tests.

For instance, under high-fertility population projections, countries experience a reduction in their total CO_2-equivalent warming budgets per capita due to increased population compared with medium-fertility population projections (Supplementary Fig. 12). This effect is more pronounced in countries with medium and low human development, as these countries' populations are more affected. However, when considering the remaining CO_2-equivalent warming budgets per capita, the impact of population projection appears to be marginal.

Here, we illustrate how national remaining (2022-2050) CO_2 warming equivalent budgets per capita vary with population projections (Supplementary Fig. 13). Generally, the disparities between country groups are normally enlarged with low fertility population projection. That means, medium and low human development countries get larger budgets, and very high human development countries get fewer budgets. The disparities between country groups are normally decreased with high fertility population projection.

Supplementary Note 8: Illustration of alternative country categorization method

Here, we illustrate how total (1850-2050), historical (1850-2021), and remaining (2022-2050) CO_2 warming equivalent budgets per capita vary by country, and per category according to the World Bank income classification[2], and an alternative classification which comprises of developed countries, developing countries and least developed countries (LDCs). The classification of developed countries, developing countries, and LDCs follows the Standard Country or Area Codes for Statistical Use (M49)[3], in the version as of May 2022.

Here we categorize countries by World Bank income classification (Supplementary Fig. 14). Out of 60 high income countries, 54 are left with negative remaining budgets for the 2022-2050 period under equity Interpretation I. The ten countries with the smallest budgets include Trinidad and Tobago, Estonia, Qatar, Brunei Darussalam, Bahrain, United States, Germany, Luxembourg, Barbados and United Kingdom. These countries can be categorized into two groups: those rich in oil and gas resources, such as Qatar, Brunei Darussalam, and Bahrain, with small populations and economies heavily reliant on oil and gas exports; and developed nations with early industrialization and industries based on fossil fuels. Half (26 out of 52) of upper middle income countries maintain negative budgets, including resource-rich economies like Russia, emerging economies like China and South Africa, and transition economies like Bulgaria. Nearly all (50 out of 54) lower middle income countries have positive remaining CO_2-we budgets. These countries are primarily located in South and Southeast Asia, Africa, Central and South America, as well as the island nations of Oceania. All low income countries except for North Korea have positive remaining CO_2-we budgets above the global average.

Here we categorize countries by developed countries, developing countries and LDCs (Supplementary Fig. 15). Out of 51 developed countries, 48 are left with negative remaining budgets for the 2022-2050 period under equity Interpretation I. Typical countries include the United States (US), the United Kingdom (UK), Russia, and Japan, due to their early industrialization and fossil fuel-based industries. About two-thirds (60 out of 98) developing countries maintain positive budgets. These countries experienced later industrialization with lower historical emissions compared to developed countries. Several developing countries are among the ten countries with the lowest remaining CO_2-we emissions budget, even below most developed nations. These countries include Trinidad and Tobago, Palau, Qatar, Brunei Darussalam, and Bahrain, which mostly are nations with small populations and economies based on oil and gas extraction, resulting in extremely high historical per capita emissions. All LDCs have positive remaining CO_2-we budgets above the global average.

Supplementary Note 9: Description of data sources and data preparation process

The data sources and detailed description of data are shown in Supplementary Table 1.For 10-year bond yield, data are sourced from two datasets[4, 5] and individual web searches. Supplementary Table 2 lists the countries included in each data source and provides the corresponding web links. We first combine the information of the two formal datasets[4, 5]. For countries with data available from both datasets, we adopt the average value. For a country with no data in both datasets, we conduct web searches if the country is amongst the top 30 most populous countries globally or a top 30 current emitter worldwide. In cases where no 10-year bond yield data was available, we used 3-year bond yield or 10+ years yield instead. Data of eight additional countries are derived in this way. For the remaining nations with missing data, we fill values based on the average value within their respective groups: developed countries, developing countries, or LDCs. For fossil fuel sales, national historical cumulative fossil fuel sales are calculated by summing the products of the prices and production volumes of coal, oil, and gas, aggregated cumulatively from 1900 to 2016. Coal, oil, and gas production data from 1900 to 2016 are derived from Ourworldindata[6] which combines information from the Energy Institute Review of World Energy[7] and ref.[8]. Coal, natural gas price from 1850 to 2020 are derived from D. S. Jacks[9]. Historical crude oil prices from 1861 to 2023 are derived from ChartsBin[10] showing data from the BP Statistical Review of World Energy[11, 12].

Supplementary Figures

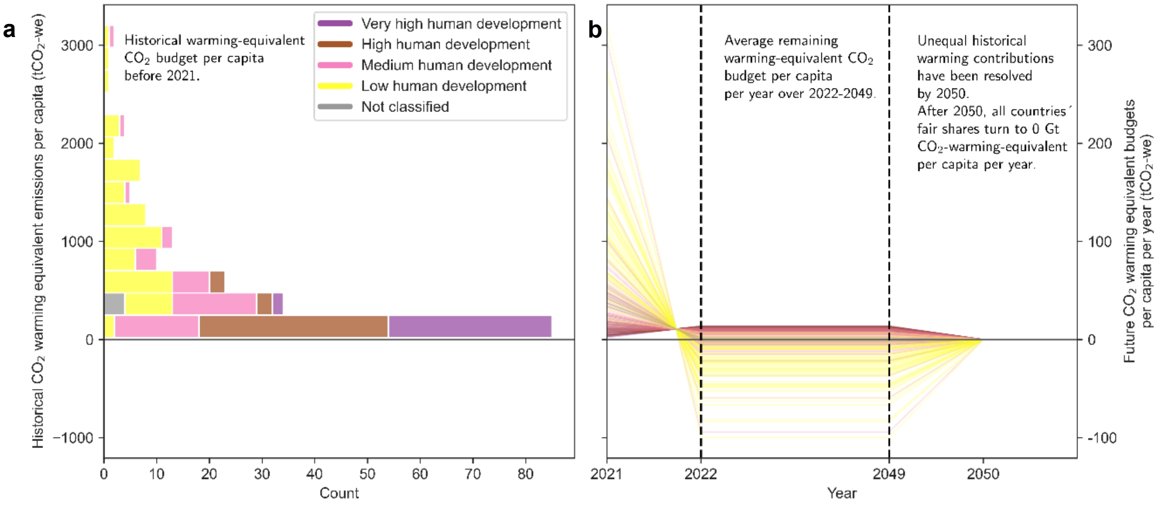

Supplementary Fig. 1 Conceptual schematic of the resolution of countries' unequal historical warming contributions by 2050 through fair warming allocations. a Distribution of national historical CO_2 warming equivalent emissions per capita by 2021. The count of countries is shown on the x-axis. The historical emissions per capita by 2021 are shown on the y-axis. **b** Change of national CO_2 warming equivalent emissions per capita over time. Each line represents an individual country. The leftmost part of the subfigure refers to the historical emissions by 2021, identical to **a**. The part between two dotted lines illustrates the emission budgets per capita per year between 2022 and 2049; the part on the right-hand side of the dotted line refers to emission budgets per capita in 2050; both are shown on the right y-axis.

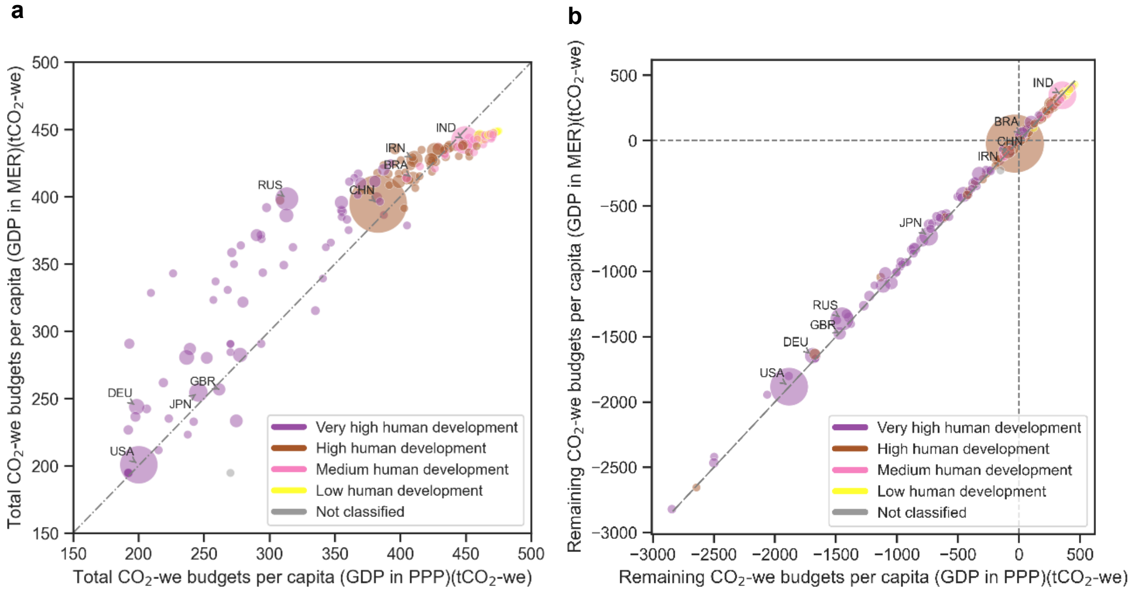

Supplementary Fig. 2 Influence of GDP definition on CO_2 warming equivalent budgets. Total (1850-2050) (**a**) and remaining (2021-2050) (**b**) CO_2 warming equivalent budgets per capita,

compared when using GDP measured in market exchange rates (MER) and purchasing power parity (PPP) as an indicator for ability to pay. The size of bubbles indicates the amount of national emissions in 2021. Very high, high, medium, and low human development countries are marked in different colours. A few major economies are labelled with their ISO3 codes.

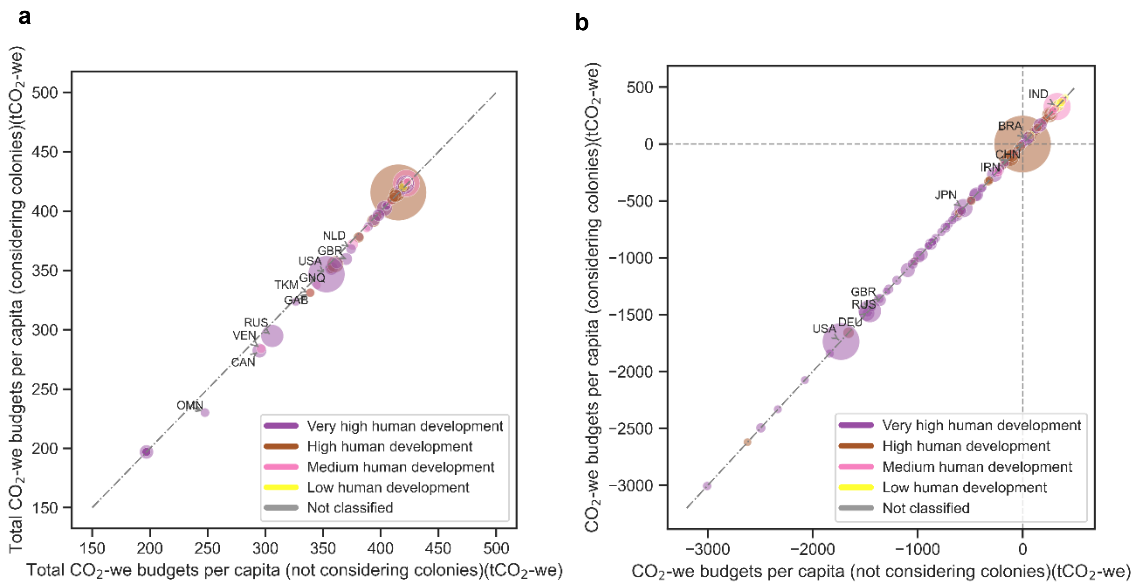

Supplementary Fig. 3 Influence of apportioning fossil fuel sales during colonial occupation to colonizers. Total (1850-2050) (**a**) and remaining (2022-2050) (**b**) CO_2 warming equivalent budgets per capita, compared between using per capita fossil fuel sales with and without redistribution of the colonies. Both allocations reflect our Interpretation III, which is based on equality, polluter pays and beneficiary pays principles, using fossil fuel sales as the key indicator. They differ in whether fossil fuel sales are redistributed to the colonies (y-axis) or not (x-axis). The size of the bubbles indicates the amount of national emissions in 2021. Very high, high, medium, and low human development countries are marked in different colours.

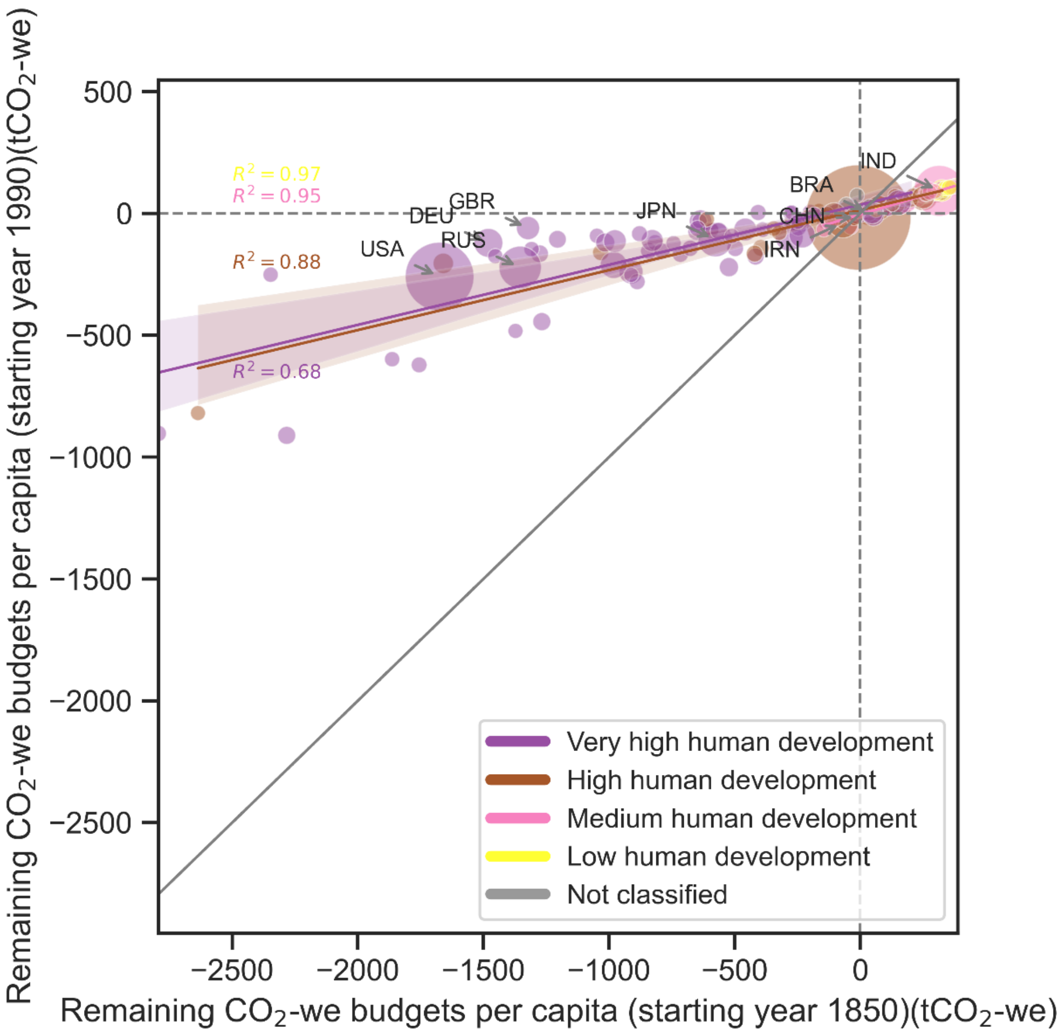

Supplementary Fig. 4 Remaining (2021-2050) CO_2 warming equivalent budgets per capita, compared between different starting years of historical warming contribution. Both allocations reflect our Interpretation I, which is based on the equality and polluter pays principles. They differ in terms of the starting years for historical warming contributions, 1850 for the x-axis, and 1990 for the y-axis. The size of the bubbles indicates the amount of national emissions in 2021. Very high, high, medium, and low human development countries are marked in different colours. Regression analysis is conducted for each country group, with R^2 values provided in the respective colours.

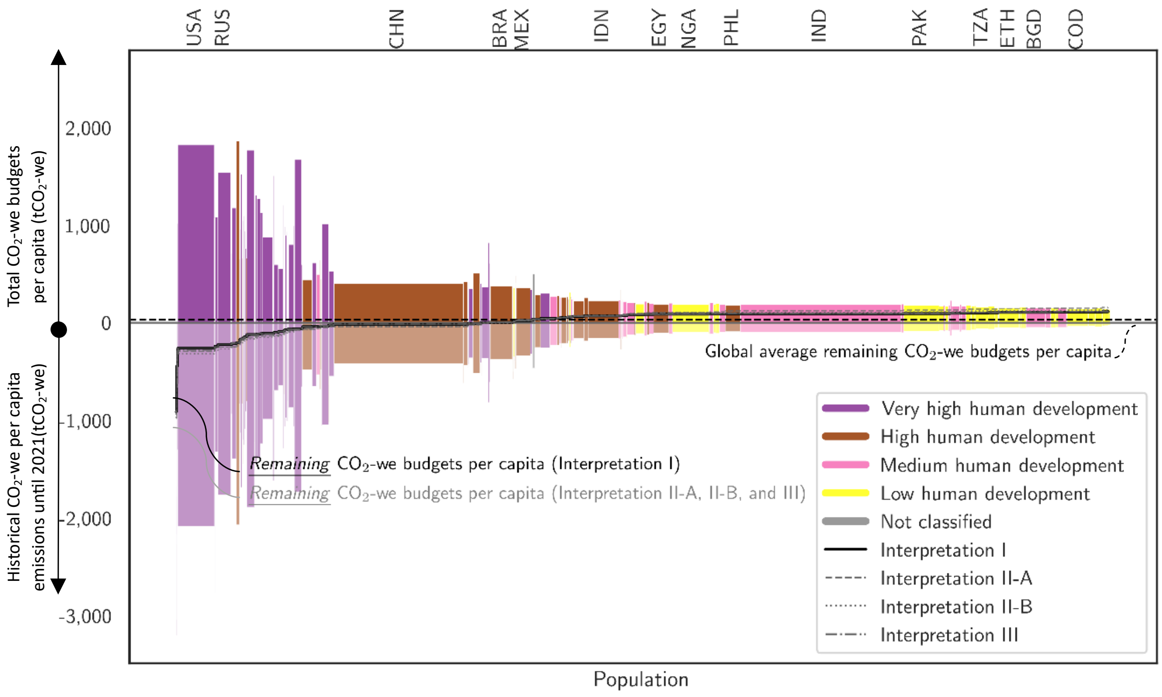

Supplementary Fig. 5 Total (1850-2050), historical (1850-2021), and remaining (2022-2050) CO_2 warming equivalent budgets per capita by country (starting year of historical warming contribution of 1990). Countries are ranked according to their remaining per capita national budgets, under the central case which considers a historical warming contribution starting in 1850 and a global warming target of 1.5°C. The height of bars above the x-axis represents the total CO_2-we budgets per capita under equity Interpretation I that considers the equality and polluter-pays principles. The height of the bars below the x-axis represents the historical consumed CO_2-we budgets per capita. The width of the bars represents a country's population in 2050. The step lines represent the remaining CO_2-we budgets per capita per country with the black line referring to Interpretation I and the grey lines referring to Interpretation II-A, II-B, and III. Very high, high, medium, and low human development countries are marked in different colours.

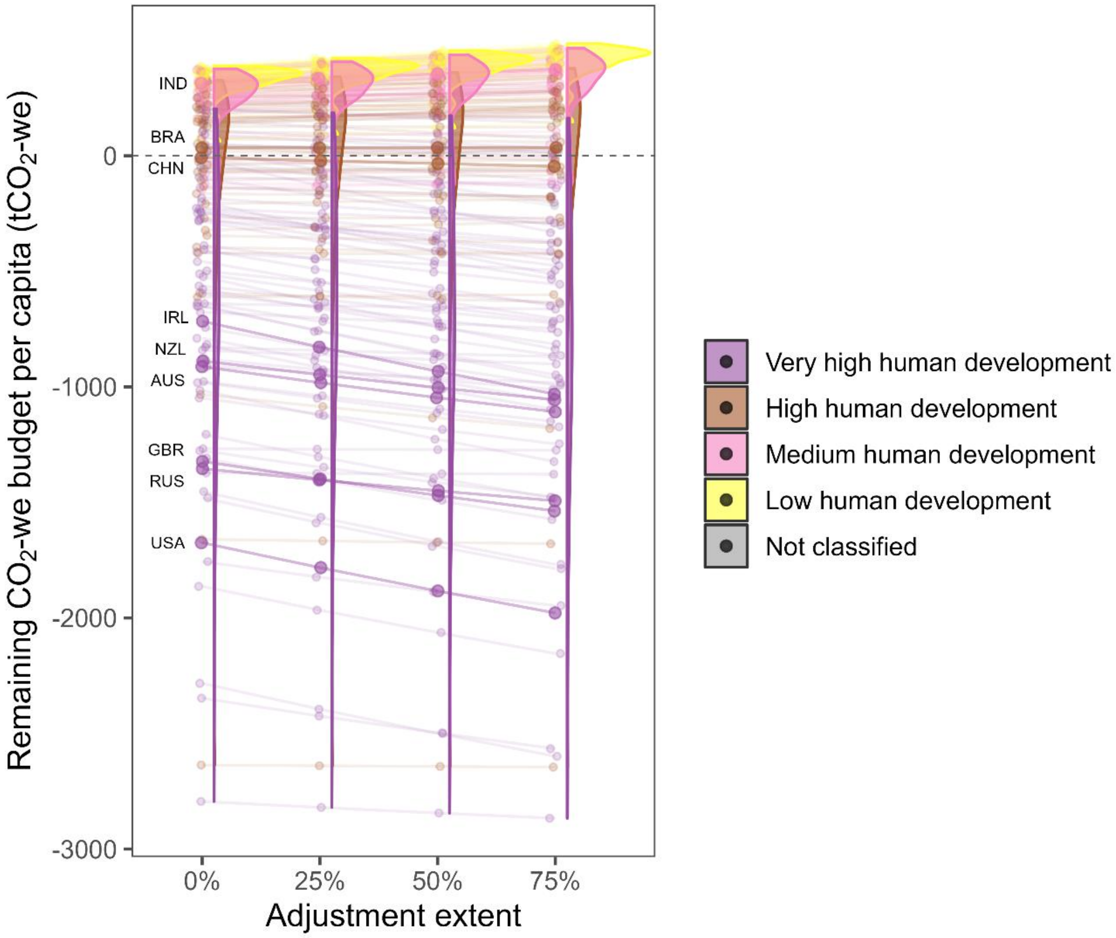

Supplementary Fig. 6 The uncertainty in remaining (2022-2050) CO_2 warming equivalent budgets for major countries, with various maximum adjustment extent of driving indicators. The x-axis shows different adjustment extents. “Adjustment extent” refers to the degree to which a country’s allocation is changed as a function of differences in driving indicators. Connected points represent data from the same country, showing how a countries allocation and relative position changes as adjustment extent is changed. The overlapped density plots depict the distribution of remaining budgets by country group.

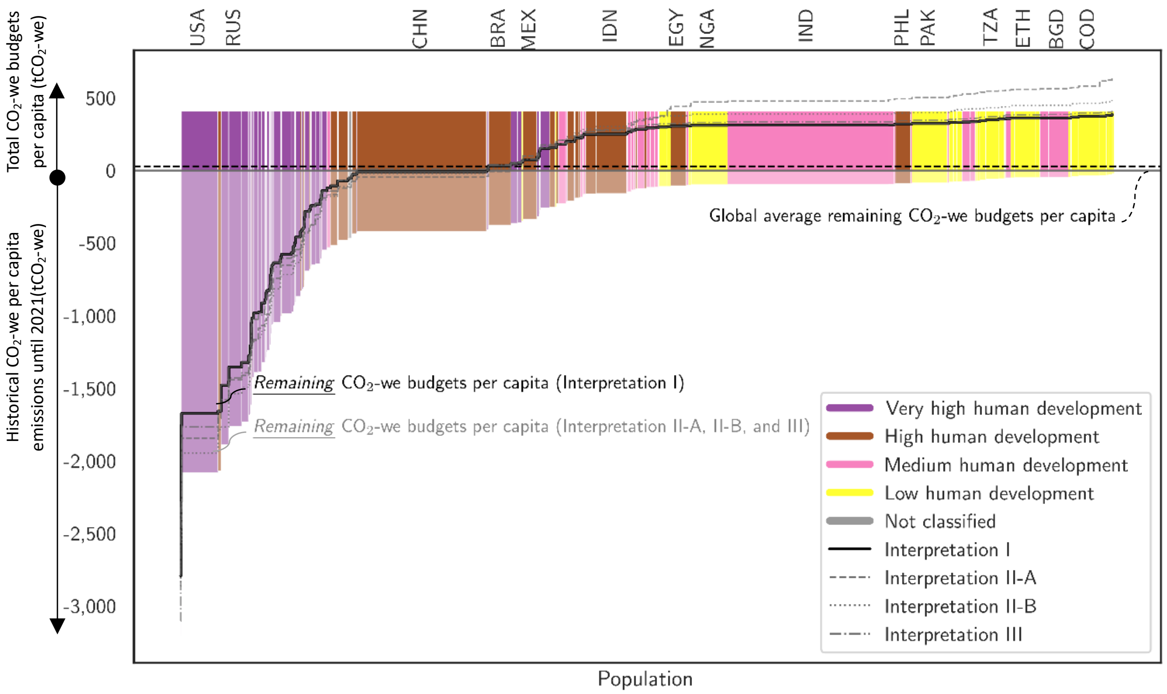

Supplementary Fig. 7 Total (1850-2050), historical (1850-2021), and remaining (2022-2050) CO_2 warming equivalent budgets per capita by country (adjustment extent of 75%). Countries are ranked according to their remaining per capita national budgets, under a sensitivity analysis case which considers the maximum adjustment extent of driving indicators to be 75%. The height of bars above the x-axis represents the total CO_2-we budgets per capita under equity Interpretation I that considers the equality and polluter-pays principles. The height of the bars below the x-axis represents the historical consumed CO_2-we budgets per capita. The width of the bars represents a country's population in 2050. The step lines represent the remaining CO2-we budgets per capita per country with the black line referring to interpretation I and the grey lines referring to Interpretation II-A, II-B, and III. Very high, high, medium, and low human development countries are marked in different colours.

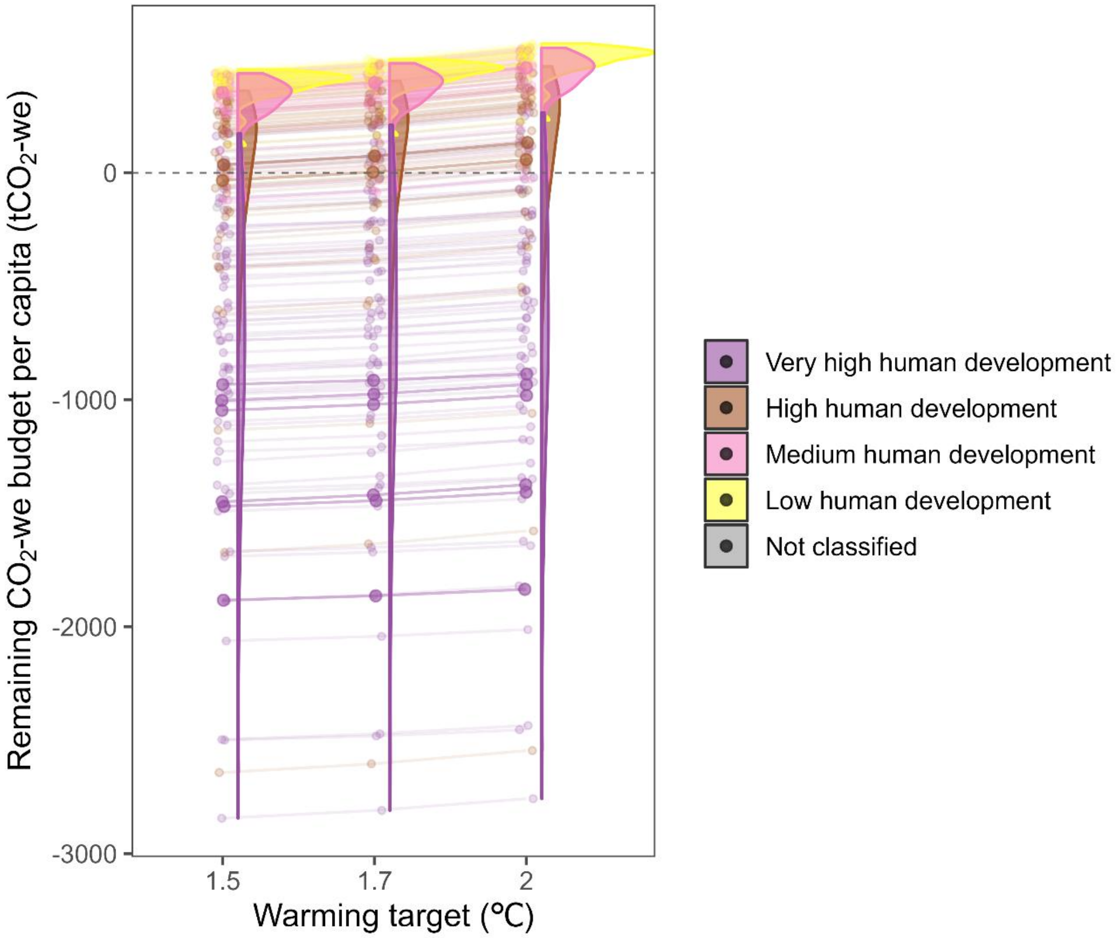

Supplementary Fig. 8 The uncertainty in remaining (2022-2050) CO_2 warming equivalent budgets for major countries, with various maximum adjustment extent of driving indicators. The x-axis shows different desired peak global warming target. Connected points represent data from the same country, showing how a countries allocation and relative position changes as desired peak global warming target is changed. The overlapped density plots depict the distribution of remaining budgets by country group.

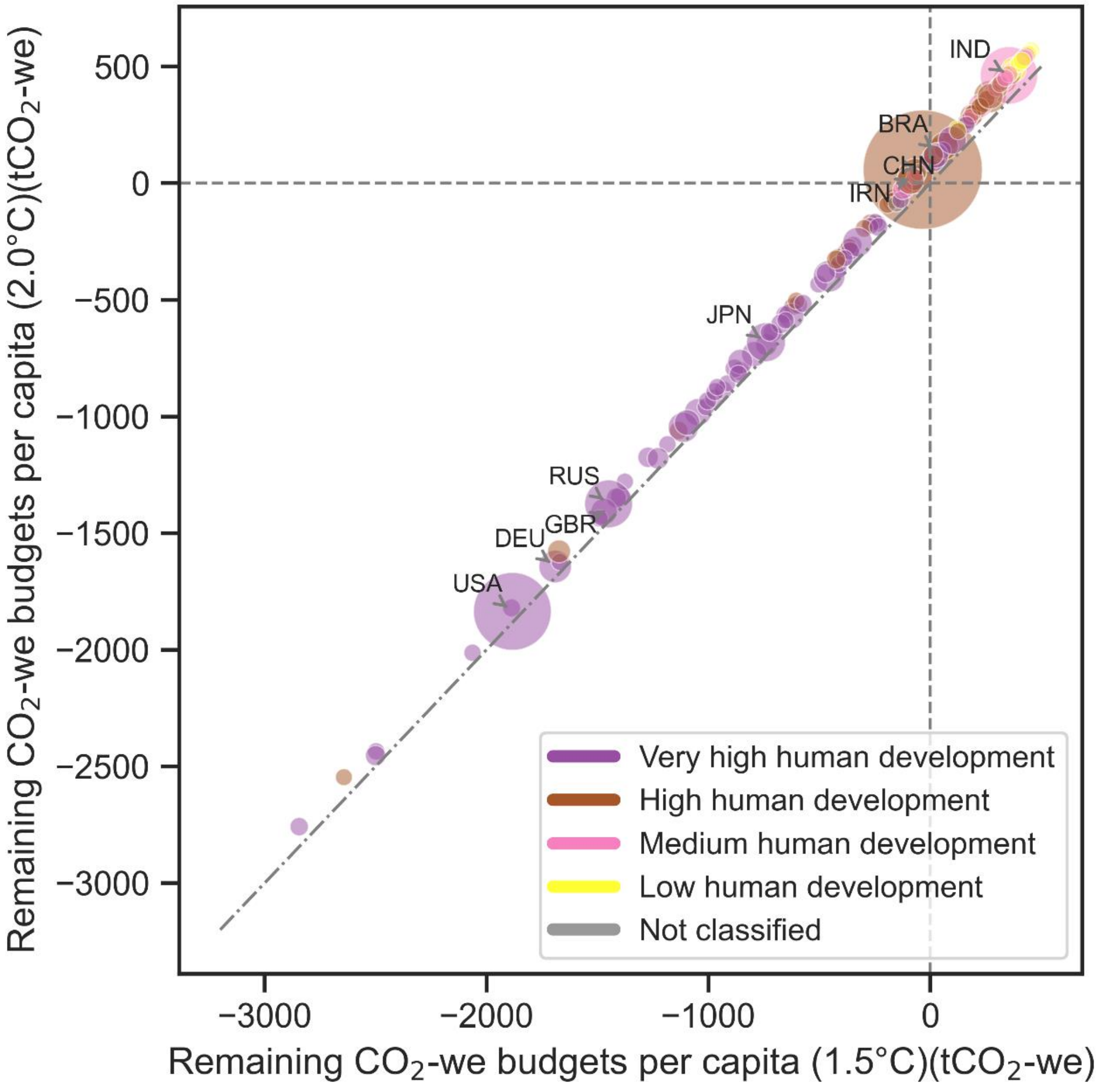

Supplementary Fig. 9 Remaining (2022-2050) CO_2 warming equivalent budgets per capita, compared between different desired peak global warming target. Both allocations adhere to the equity Interpretation II-B. They differ in terms of the desired peak global warming target, 1.5℃ for the x-axis, and 2℃ for the y-axis. The size of the bubbles indicates the amount of national emissions in 2021. Very high, high, medium, and low human development countries are marked in different colours.

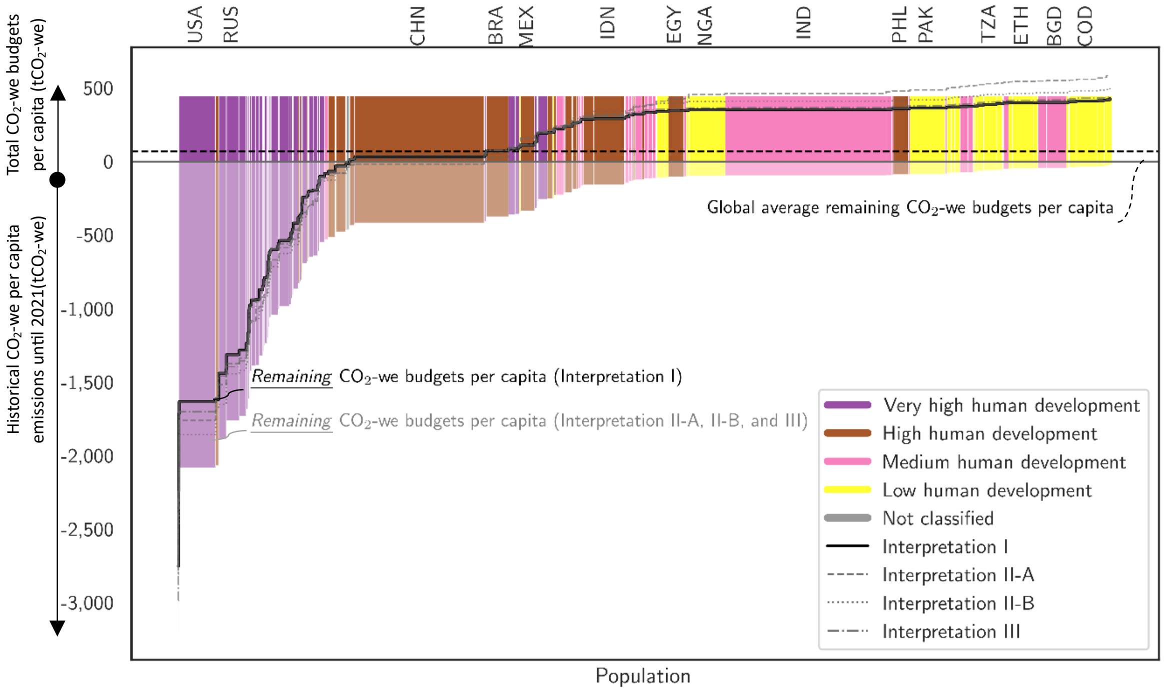

Supplementary Fig. 10 Total (1850-2050), historical (1850-2021), and remaining (2021-2050) CO_2 warming equivalent budgets per capita by country (desired peak global warming target of 1.7℃). Countries are ranked according to their remaining per capita national budgets, under a sensitivity analysis case which considers a historical warming contribution starting in 1850 and a global warming target of 1.7°C. The height of bars above the x-axis represents the total CO_2-we budgets per capita under equity Interpretation I that considers the equality and polluter-pays principles. The height of the bars below the x-axis represents the historical consumed CO_2-we budgets per capita. The width of the bars represents a country's population in 2050. The step lines represent the remaining CO_2-we budgets per capita per country with the black line referring to Interpretation I and the grey lines referring to Interpretation II-A, II-B, and III. Very high, high, medium, and low human development countries are marked in different colours.

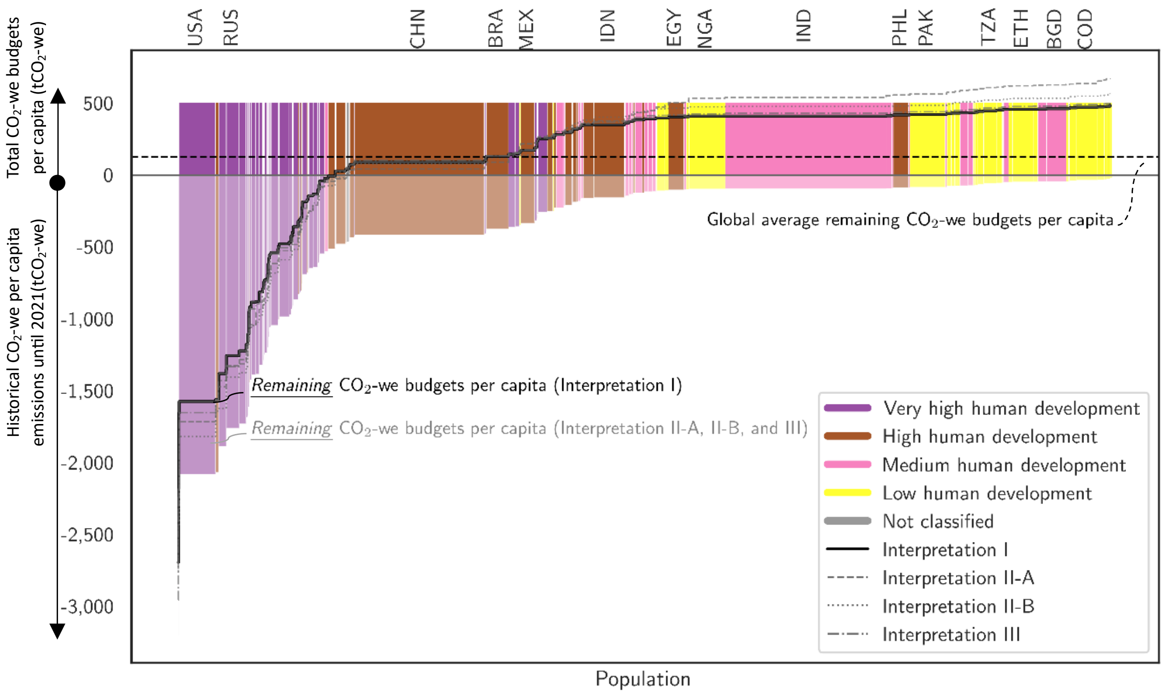

Supplementary Fig. 11 Total (1850-2050), historical (1850-2021), and remaining (2021-2050) CO_2 warming equivalent budgets per capita by country (desired peak global warming target of 2℃). Countries are ranked according to their remaining per capita national budgets, under a sensitivity analysis case which considers a historical warming contribution starting in 1850 and a global warming target of 2°C. The height of bars above the x-axis represents the total CO_2-we budgets per capita under equity Interpretation I that considers the equality and polluter-pays principles. The height of the bars below the x-axis represents the historical consumed CO_2-we budgets per capita. The width of the bars represents a country's population in 2050. The step lines represent the remaining CO_2-we budgets per capita per country with the black line referring to Interpretation I and the grey lines referring to Interpretation II-A, II-B, and III. Very high, high, medium, and low human development countries are marked in different colours.

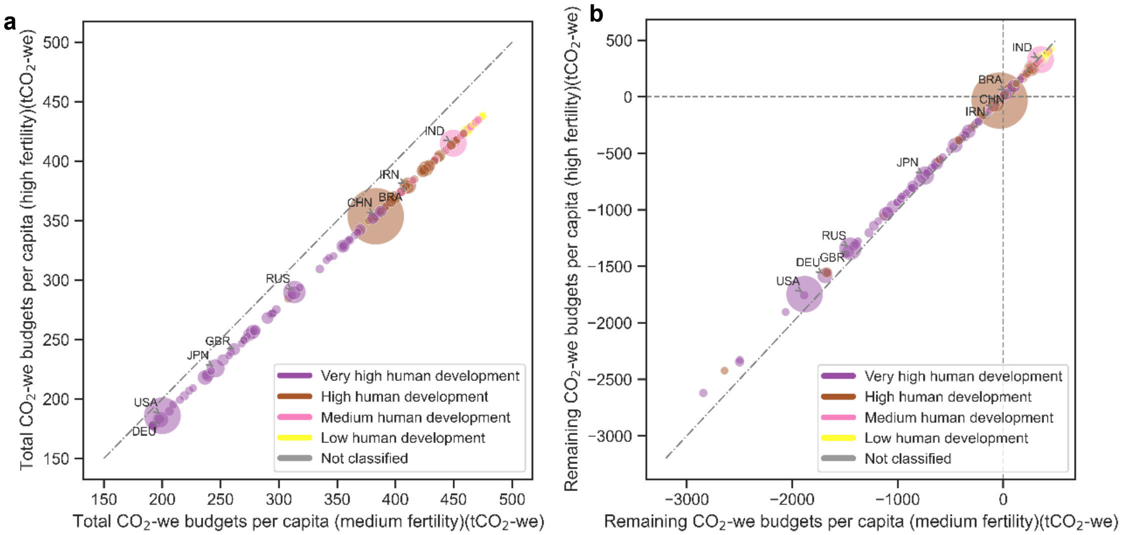

Supplementary Fig. 12 Influence of population projection on CO_2 warming equivalent budgets. Total (1850-2050) (a) and remaining (2022-2050) (b) CO_2 warming equivalent budgets per capita, compared when using population projection under both medium and high fertility scenarios. All allocations adhere to the equity Interpretation I. The size of bubbles indicates the amount of national emissions in 2021. Very high, high, medium, and low human development countries are marked in different colours.

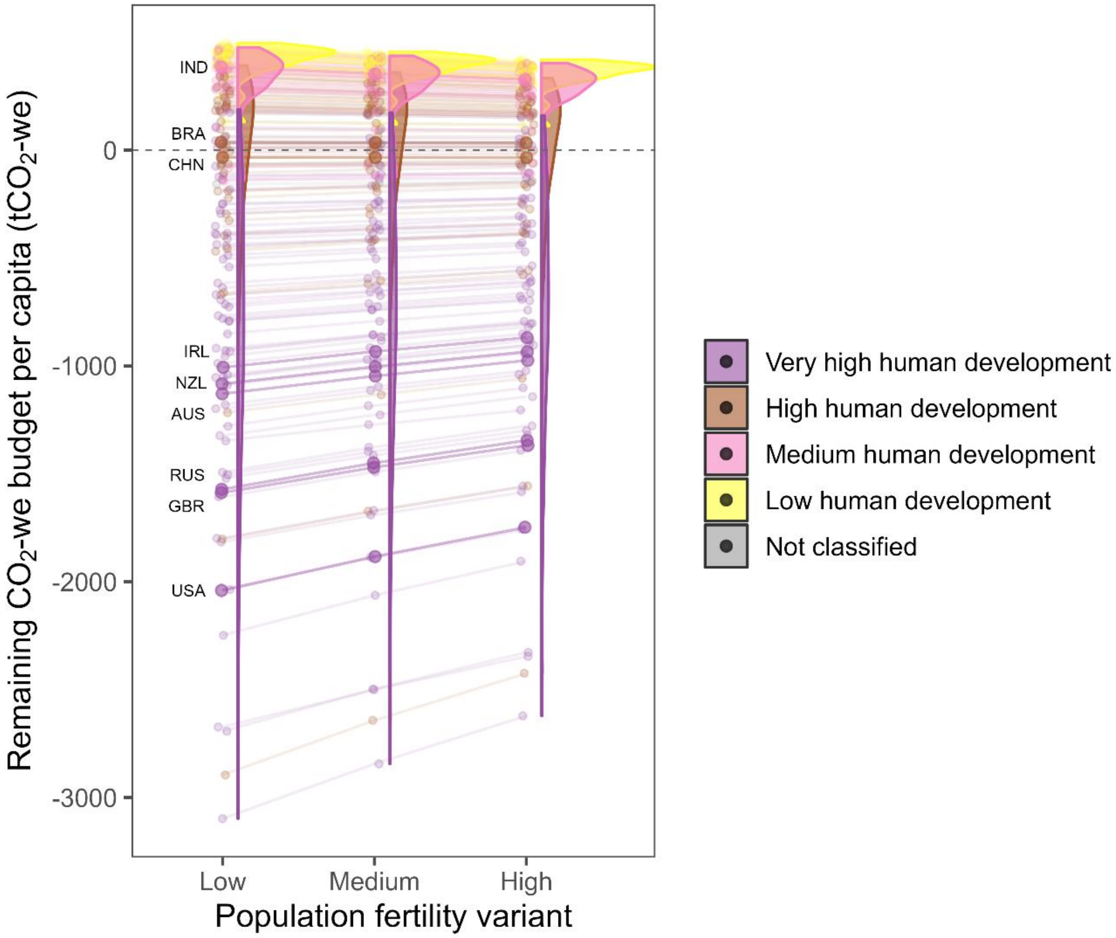

Supplementary Fig. 13 The uncertainty in remaining (2022-2050) CO_2 warming equivalent budgets for major countries, with various population projections. All allocations adhere to the equity Interpretation I. The x-axis shows different population projections due to fertility. Connected points represent data from the same country, showing how a country's budget changes as population projection is changed. The overlapped density plots depict the distribution of remaining budgets by country group.

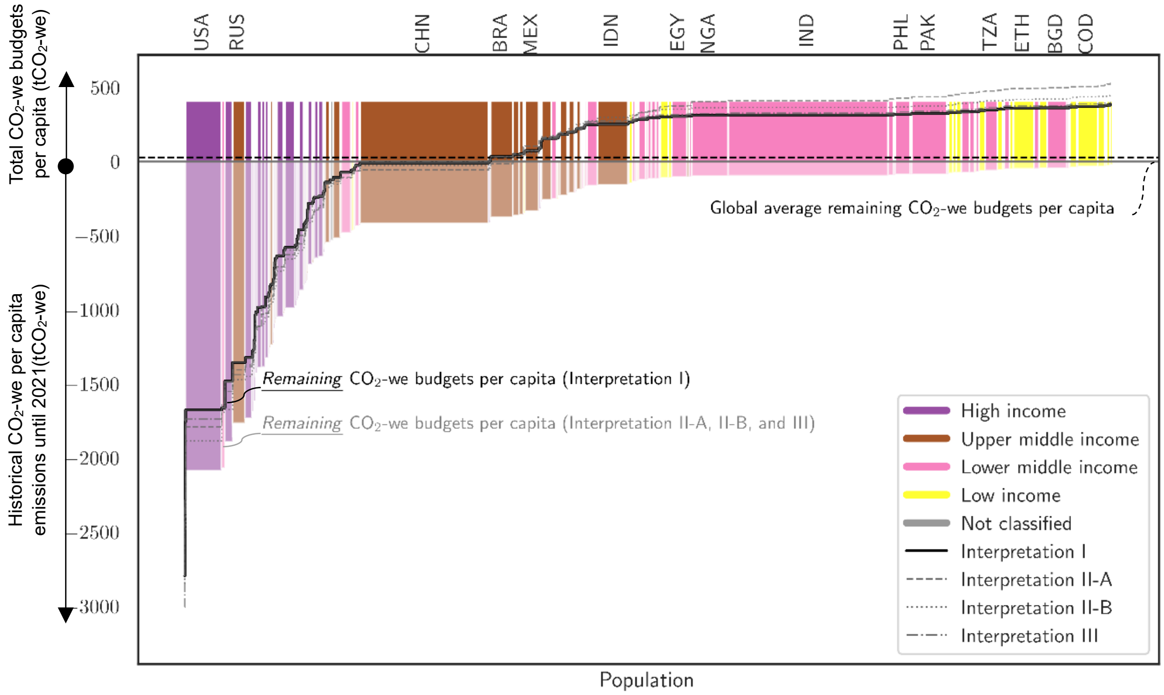

Supplementary Fig. 14 Total (1850-2050), historical (1850-2021), and remaining (2022-2050) CO_2 warming equivalent budgets per capita by country. Countries are ranked according to their remaining per capita national budgets, under the central case which considers a historical warming contribution starting in 1850 and a global warming target of 1.5°C. The height of bars above the x-axis represents the total CO_2-we budgets per capita under equity Interpretation I that considers the equality and polluter-pays principles. The height of the bars below the x-axis represents the historical consumed CO_2-we budgets per capita. The width of the bars represents a country's population in 2050. The step lines represent the remaining CO_2-we budgets per capita per country with the black line referring to Interpretation I and the grey lines referring to Interpretation II-A, II-B, and III. High, upper middle, lower middle and low income countries are marked in different colours.

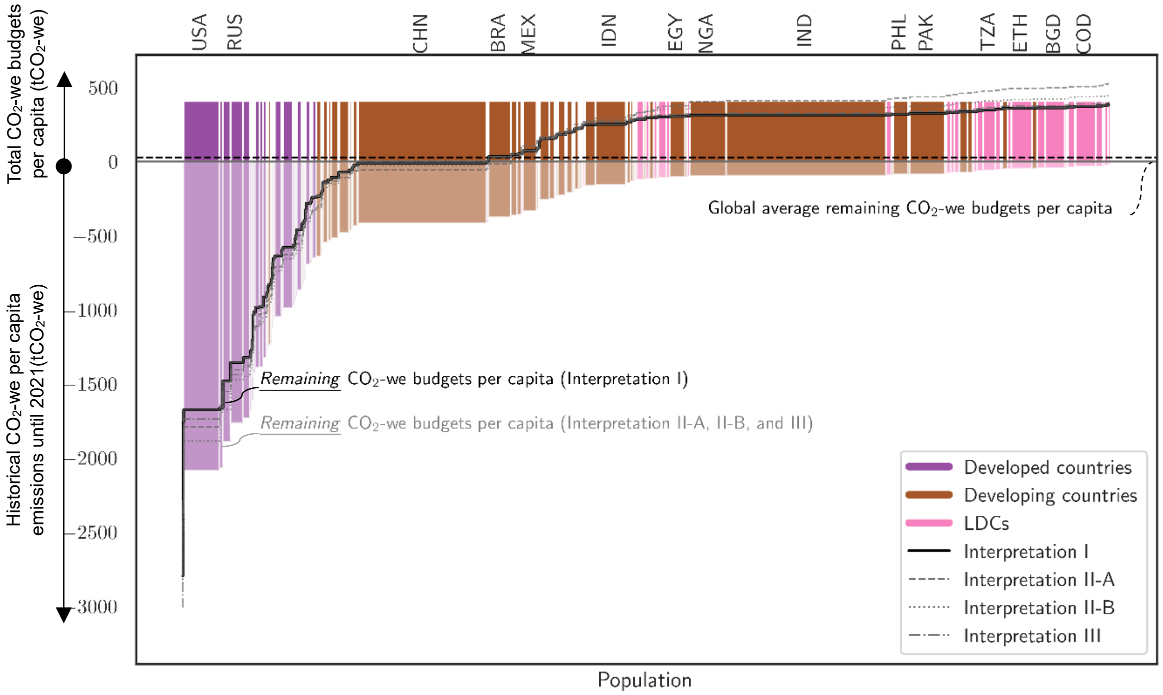

Supplementary Fig. 15 Total (1850-2050), historical (1850-2021), and remaining (2022-2050) CO_2 warming equivalent budgets per capita by country. Countries are ranked according to their remaining per capita national budgets, under the central case which considers a historical warming contribution starting in 1850 and a global warming target of 1.5°C. The height of bars above the x-axis represents the total CO_2-we budgets per capita under equity Interpretation I that considers the equality and polluter-pays principles. The height of the bars below the x-axis represents the historical consumed CO_2-we budgets per capita. The width of the bars represents a country's population in 2050. The step lines represent the remaining CO_2-we budgets per capita per country with the black line referring to Interpretation I and the grey lines referring to Interpretation II-A, II-B, and III. Developed countries, developing countries and least developed countries (LDCs) are marked in different colours.

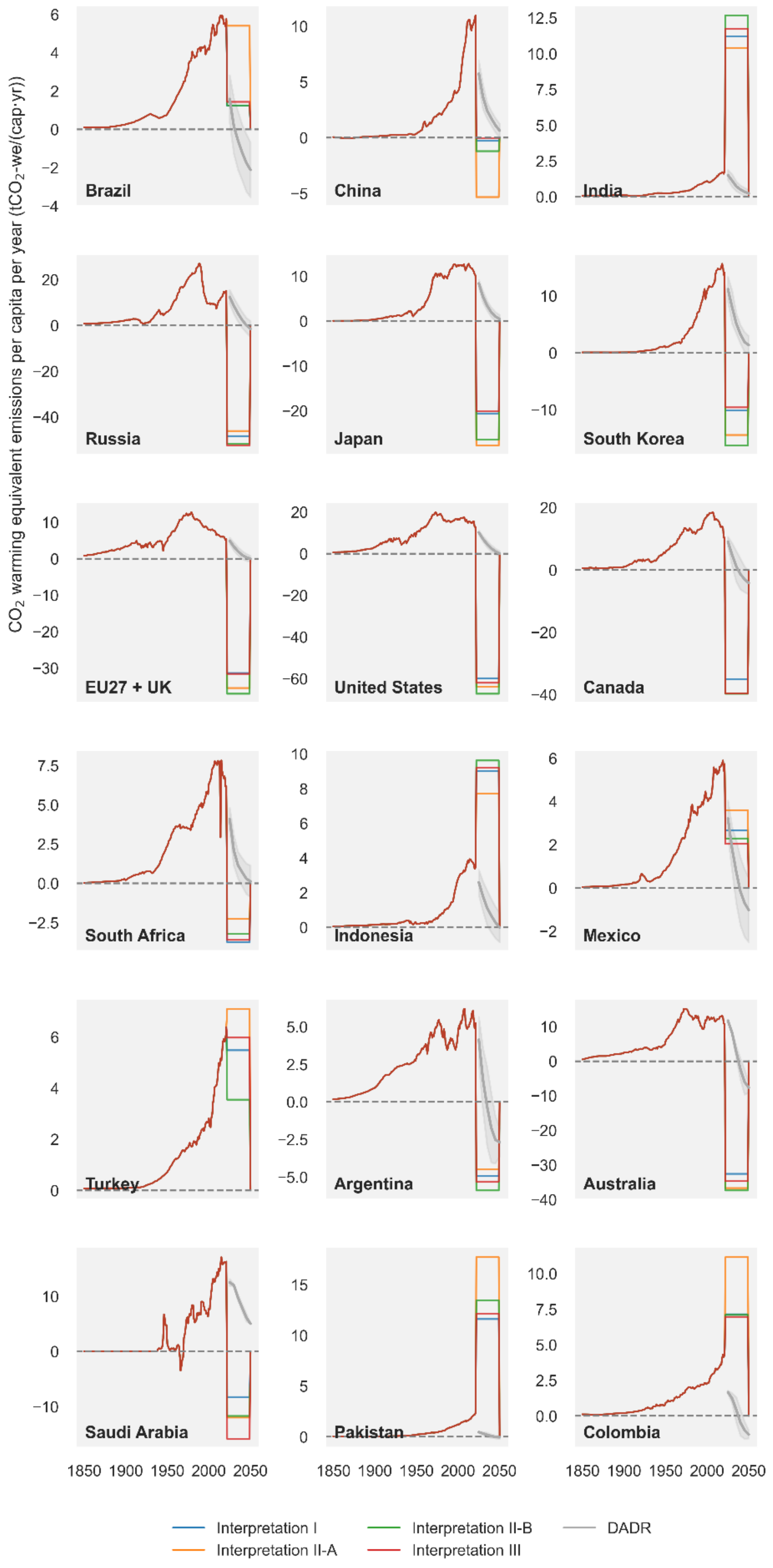
CO2 warming equivalent emissions per capita per year (tCO2-we/(cap·yr))
Brazil
China
India
Russia
Japan
South Korea
EU27 + UK
United States
Canada
South Africa
Indonesia
Mexico
Turkey
Argentina
Australia
Saudi Arabia
Pakistan
Colombia
1850 1900 1950 2000 2050
Interpretation I
Interpretation II-A
Interpretation II-B
Interpretation III
DADR

Supplementary Fig. 16 Annual fair CO_2 warming equivalent emission budgets against deepest available domestic reduction (DADR) pathways. For the period 1850-2021, the historical CO_2-we GHG emissions per capita data are shown in a single line for each country. For the period 2022-2050, the annual budgets per capita are shown in coloured lines according to the different equity interpretations. For the period 2022-2050, shaded grey areas additionally show the DADR pathways from the IPCC Sixth Assessment Report database C1 category for each country or region, including CO_2 emissions only. The solid grey line within these areas indicates the median of the DADR pathways. EU27 + UK is short for the European Union (27 countries) and UK. EU27+UK countries are treated as individual entities through the fair allocation process, and are combined only for illustrative purposes.

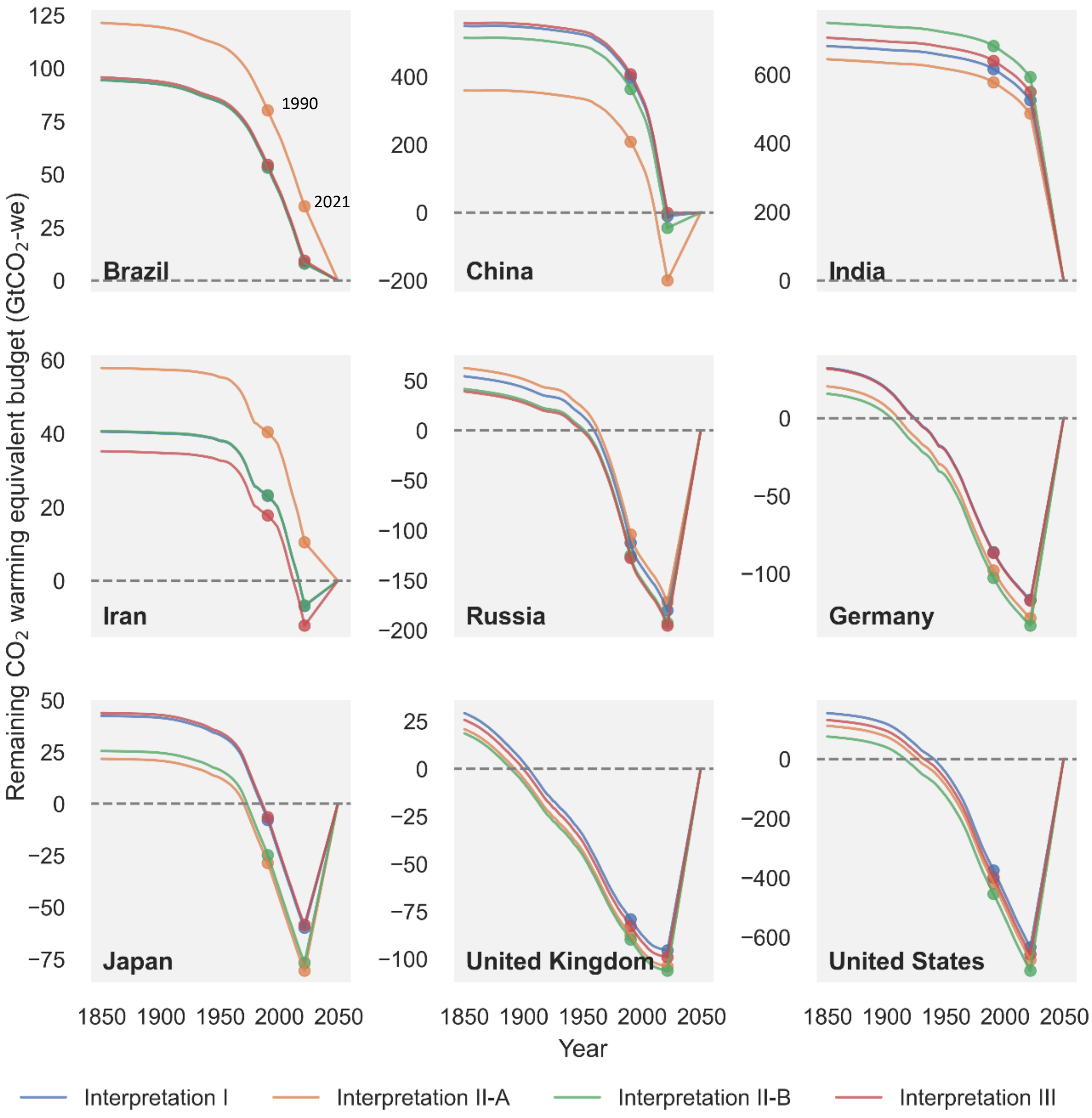

Supplementary Fig. 17 The depletion of remaining CO_2 warming equivalent emission budgets over time. This figure differs from Fig. 3, with the x-axis extended to 2050 to illustrate a stylized example of fair allocation. During the period 2022–2049, countries adhere to fair allocations

with equal annual quotas. By 2050, all countries return to zero CO_2-we emissions. These evolutions for the 2022–2049 are illustrative and do not represent technically feasible emissions evolutions.

Supplementary Tables

Supplementary Table 1 Data sources used in this study

Data	Data details	Time	Source
GHG emissions	National historical GHG emissions, excluding Agriculture, Forestry and Other Land Use and international aviation and shipping sectors, HISTCR scenario	1850-2021	[13, 14]
Aerosol emissions	Global historical aerosol emissions, averaging over all scenarios in IPCC AR6 database	1850-2019	[15]
Population	National population as of 1 July, United Nations medium scenario projection	2050	[16]
10-year bond yield	10-year government bond yield	Around 2023	[4, 5], web searches
GDP	Total gross domestic product (GDP), purchasing power parity (PPP)	2021	[17]
	GDP, market exchange rates (MER)	2021	[18]
Fossil fuel sales	National historical fossil fuel sales, including coal, oil, and gas	1900-2016	[6, 9, 10]
Deepest available domestic reduction (DADR) pathways	National emission pathways following the target of limiting warming to 1.5°C (>50%) with no or limited overshoot (IPCC AR6 database C1 category)	2010-2050	[15]

Supplementary Table 2 Data sources for 10-year bond yield values per country

Country (or region)	Source
Japan, Switzerland, Thailand, Germany, Sweden, Denmark, Netherlands, China, France, Ireland, Finland, Austria, Belgium, Jordan, Singapore, Portugal, Canada, Norway, Slovakia, South Korea, Spain, Malta, Slovenia, United Kingdom, Australia, Croatia, United States, Malaysia, Israel, Cyprus, Morocco, Greece, Italy, Vietnam, Latvia, Lithuania, New Zealand, Czech Republic, Bulgaria, Mauritius, Bahrain, Chile, Philippines, Namibia, Poland, Indonesia, Botswana, Serbia, Iceland, India, Romania, Perù, Bangladesh, Hungary, Mexico, South Africa, Turkey, Russia, Colombia, Kazakhstan, Brazil, Nigeria, Kenya, Uganda, Pakistan, Ukraine, Egypt, Sri Lanka, Zambia	http://www.worldgovernmentbonds.com/[4]
United Kingdom, Germany, Russia, Italy, France, Switzerland, Turkey, Slovakia, Slovenia, Spain, Sweden, Latvia, Lithuania, Netherlands, Norway, Poland,	https://tradingeconomics.com/bonds[5]

Portugal, Romania, Greece, Hungary, Iceland, Ireland, Austria, Belgium, Bulgaria, Croatia, Czech Republic, Denmark, Finland, United States, Brazil, Canada, Chile, Colombia, Mexico, Venezuela, Japan, India, China, Singapore, South Korea, Thailand, Vietnam, Indonesia, Israel, Malaysia, Pakistan, Philippines, Qatar, Australia, New Zealand, South Africa, Zambia, Kenya, Nigeria	
Ethiopia	https://www.reuters.com/article/ethiopia-eurobonds-idUSL6N0TO2FB20141204
Tanzania	https://www.tanzaniainvest.com/tourism/economic-review-2022
Iran	https://en.wikipedia.org/wiki/Banking_and_insurance_in_Iran
Iraq	https://www.iraq-businessnews.com/2017/08/03/huge-demand-for-iraqi-govt-bonds/
Angola	https://www.reuters.com/article/angola-bonds-idUSL8N12Z3VK20151104
Saudi Arabia	https://research.ftserussell.com/Analytics/FactSheets/Home
Kuwait	https://www.ceicdata.com/en/kuwait/treasury-bills-and-bonds-rate-average/treasury-bills--bonds-rate-10-years
Albania	http://www.xinhuanet.com/english/2019-01/15/c_137743740.htm

Supplementary Table 3 Normative, methodological and physical factors considered and their parameter settings

Category	Parameters / metrics	Default setting	Alternative settings
Normative uncertainty	Starting year of historical contributions	1850	1990
Physical uncertainty	Desired peak global warming target	1.5°C	1.7°C, and 2°C
	Estimation of the aerosol masking effect at the time of peak warming	50% percentile	10%, 25%, 33%, 66%, 75%, and 90% percentile
Methodological uncertainty	Maximum degree to which a country's allocation is changed as a function of differences in driving indicators	50%	25%, and 75%

Supplementary Table 4 Warming masked by aerosol at various percentiles and consistent with a set of peak levels of global warming. Negative values indicate a reduction in the total warming relative to 1850-1900 temperatures.

Peak warming (℃)	Aerosol masking effect (℃)						
	0.1 percentile	0.25 percentile	0.33 percentile	0.5 percentile	0.66 percentile	0.75 percentile	0.9 percentile
1.5	-1.06862	-0.56715	-0.45565	-0.28514	-0.15310	-0.10244	-0.00151
1.6	-1.03990	-0.54500	-0.43481	-0.27063	-0.14729	-0.09637	0.00170
1.7	-1.01119	-0.52286	-0.41398	-0.25611	-0.14149	-0.09030	0.00491
1.8	-0.98247	-0.50071	-0.39314	-0.24159	-0.13568	-0.08423	0.00812
1.9	-0.95375	-0.47857	-0.37230	-0.22708	-0.12987	-0.07816	0.01133
2	-0.92503	-0.45642	-0.35146	-0.21256	-0.12407	-0.07209	0.01455

Supplementary References

1. Semieniuk G, Ghosh J, Folbre N. Technical comment on "Fairness considerations in global mitigation investments". *Science* 2023, **380**(6646)**:** eadg5893.

2. The World Bank. World Bank Country and Lending Groups. 2024 [cited 2024-06-07]Available from: https://datahelpdesk.worldbank.org/knowledgebase/articles/906519-world-bank-country-and-lending-groups

3. United Nations Statistical Office. United Nations Standard Country Code. Series M: Miscellaneous Statistical Papers, No. 49, New York: United Nations. ST/ESA/STAT/SER.M/49. 1998 [cited 2023-09-12]Available from: https://unstats.un.org/unsd/methodology/m49/

4. World Government Bond. 10Y Bond Yield. 2023 [cited 2023-02-27]Available from: http://www.worldgovernmentbonds.com/

5. Trading Economics. Major10Y. 2023 [cited 2023-03-01]Available from: https://tradingeconomics.com/bonds

6. Hannah Ritchie, Pablo Rosado, Roser M. Fossil Fuels. 2023 [cited 2023-06-20]Available from: https://ourworldindata.org/fossil-fuels

7. Energy Institute. Statistical Review of World Energy (2024). 2024 [cited 2024-07-31]Available from: https://www.energyinst.org/statistical-review/

8. Vaclav Smil. Energy Transitions: Global and National Perspectives (Second expanded and updated edition). 2017 [cited 2024-07-31]Available from: https://vaclavsmil.com/2016/12/14/energy-transitions-global-and-national-perspectives-second-expanded-and-updated-edition/

9. Jacks DS. From boom to bust: A typology of real commodity prices in the long run. *Cliometrica* 2019, **13**(2)**:** 201-220.

10. ChartsBin statistics collector team 2014. Historical Crude Oil prices, 1861 to Present. 2023 [cited 2023-06-06]Available from: http://chartsbin.com/view/oau

11. Dudley B. BP statistical review of world energy 2018. *Energy economic, Centre for energy economics research and policy British Petroleum, Available via https://www bp com/en/global/corporate/energy-economics/statistical-review-of-world-energy/electricity html* 2018, **5**.

12. Dudley B. BP statistical review of world energy 2016. *British Petroleum Statistical Review of World Energy, Bplc editor, Pureprint Group Limited, UK* 2019.

13. Gütschow J, Pflüger M. The PRIMAP-hist national historical emissions time series v2.4.1 (1750-2021). 2023 [cited 2023-03-26]Available from: https://doi.org/10.5281/zenodo.7585420

14. Gütschow J, Jeffery ML, Gieseke R, Gebel R, Stevens D, Krapp M*, et al.* The PRIMAP-hist national historical emissions time series. *Earth System Science Data* 2016, **8**(2)**:** 571-603.

15. Byers E, Krey V, Kriegler E, Riahi K, Schaeffer R, Kikstra J*, et al.* AR6 Scenarios Database hosted by IIASA. 2022 [cited 2023-08-25]Available from: data.ece.iiasa.ac.at/ar6/

16. United Nations Department of Economic Social Affairs. World population prospects 2022: Summary of results. 2022 [cited 2023-02-25]Available from: https://www.un.org/development/desa/pd/content/World-Population-Prospects-2022

17. World Bank. GDP, PPP (current international $). 2023 [cited 2023-03-30]Available from: https://data.worldbank.org/indicator/NY.GDP.MKTP.CD

18. World Bank. GDP (constant 2015 US$). 2023 [cited 2023-03-30]Available from: https://data.worldbank.org/indicator/NY.GDP.MKTP.KD
